# Missed T8-T9 Translational Fracture with Intact Neurology: A Case Report

**DOI:** 10.31729/jnma.9126

**Published:** 2025-06-30

**Authors:** Niraj Kumar Sharma, Rishi Ram Banjade, Sabin Pokharel, Sharmila Ghimire

**Affiliations:** 1 Department of Radiology, Kathmandu Medical College and Teaching Hospital, Mahankal, Kathmandu, Nepal; 2 Kathmandu Medical College and Teaching Hospital, Mahankal, Kathmandu; 3 National Trauma Center, Mahankal, Kathmandu, Nepal

**Keywords:** *decompression*, *intact neurology*, *posterior instrumentation*, *translational fracture*

## Abstract

Translational fracture of the thoracic spine, though rare due to its stability, is often associated with spinal cord injury and neurological deficits. Few cases present with intact neurology. We report a case of a 28-year-old woman who, after a road traffic accident, had persistent back pain with intact neurology. The initial chest radiograph showed a subtle deformity that was missed, likely because a lateral view was not done, and the patient was discharged. Persistent back pain led to CT imaging, revealing a T8-T9 translational fracture, left 4th and 5th posterior rib fractures near the costovertebral junction, and a T7 transverse process fracture. MRI confirmed these findings. She underwent posterior instrumentation and decompression, with symptom relief, and remained asymptomatic on follow-up. Translational thoracic spine fractures, typically associated with neurological deficits, can be missed when neurology is intact. Timely imaging and intervention are crucial for optimal outcomes.

## INTRODUCTION

Translational fractures in spine are caused by torsional or rotational forces. They are unstable injuries commonly associated with neurological deficits.^1^ The thoracic spine is usually stable because of its alignment and support of the thoracic rib cage.^2^ The spinal canal is narrowest in the thoracic spine; therefore, any compression or injury in the thoracic spine leads to spinal cord injury, resulting in a severe neurological deficit. Only a few cases of fracture at the thoracic spine with no or minimal neurological deficit are reported.^3-5^ In such cases, the injury may go unrecognized.

Here, we present a case scenario of a female with a translational fracture of the thoracic spine and associated rib fractures with no neurological deficit.

## CASE PRESENTATION

A 28-year-old woman with a history of a road traffic accident involving a four-wheeler was initially managed at a local hospital near her residence, where her only chief complaint was back pain. There was no history of loss of consciousness, vomiting, bleeding, or external injuries noted at that time. The patient had no neurological deficits, chronic illnesses, or significant medical problems. She received pain medications, but her back pain persisted. Despite her symptoms, she was mobilized during this period.

**Figure 1:**
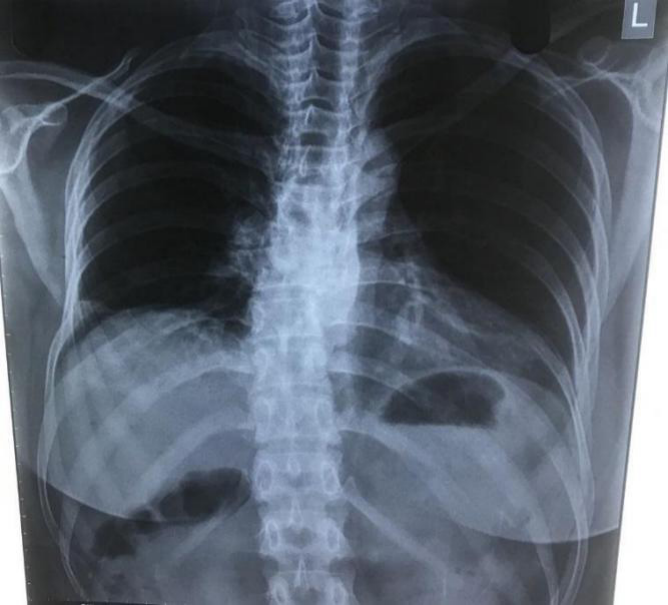
Chest X-ray posteroanterior (PA) view showing deformity at T8-T9 level along with the posterior aspect of the 4th and 5th ribs fracture near costovertebral junction.

**Figure 2:**
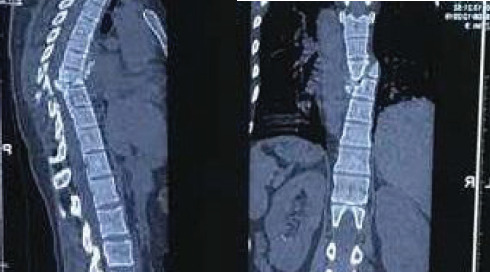
Computed tomography scan of the spine (coronal and sagittal section) showing fracture at T8-T9 level.

Since the patient was ambulating normally with intact neurological examination findings, during her initial visit a detailed spinal palpation for tenderness was not performed or documented. A chest X-ray in the posteroanterior view (Figure 1) was performed, which showed some deformity at the thoracic spine at the T8-T9 level. However, this finding was missed, likely because lateral chest X-ray views were not performed initially and the patient was discharged. Although she was able to mobilize and perform daily activities, her back pain did not resolve. One month later, with persistent back pain as her chief complaint, she revisited the same hospital. A CT scan of the spine (Figure 2) revealed a translational fracture at the T8-T9 level, classified as a Type C injury according to the AO Spine Classification System and as a three-column injury (Type C) under the Denis Three-Column Classification System. Additionally, fractures were identified in the posterior aspects of the left 4th and 5th ribs near the costovertebral junction, as well as the left T7 transverse process(Figure 1, 4). Due to the posterior location of the rib fractures, the patient did not report chest pain. Following these imaging findings, the patient was managed conservatively with Knight-Taylor brace for support and was referred to our center for further evaluation and management.

At our outpatient department, the patient presented walking independently after one month of injury, using a Knight-Taylor brace for support.

**Figure 3:**
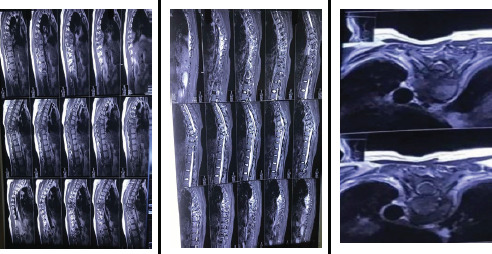
A) T1-weighted MRI sagittal section. B) T2-weighted MRI sagittal section C) The transverse section at the T8 and T9 levels demonstrates that pedicle fractures cause a separation between the posterior and middle columns, which prevents cord injury by maintaining the integrity of the spinal canal.

She was admitted to our hospital for further care. On physical examination, there was marked tenderness over the thoracic spine at the T7 to T9 levels, accompanied by a kyphotic deformity in the thoracic region measuring approximately 30 degrees, suggestive of underlying structural instability. Neurological examination revealed no sensory deficits in the T8-T9 dermatomal distribution. There was no numbness, tingling, or dysesthesia reported in the upper abdominal or lower thoracic regions. Motor strength, assessed using the modified Medical Research Council (MRC) grading scale, was 5/5 in both upper and lower limbs. Anal reflexes were intact, and there were no abnormalities in bowel or bladder habits, indicating preserved autonomic function. Local abdominal reflexes were present and symmetric. All deep tendon reflexes were within normal limits, and no pathological reflexes were elicited. An MRI of the spine was performed at our center (Figure 3), confirming the continuity of the spinal cord.

**Figure 4:**
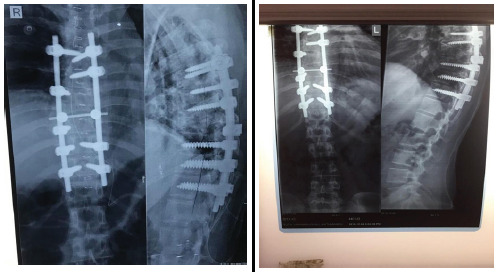
A) Post-operative 2^nd^ month, thoracic spine X-ray (anteroposterior and lateral view). B) Post-operative 6th month, anteroposterior and lateral view radiographs of thoracic spine.

The patient was prescribed medications for symptomatic relief, and surgery was planned under general anesthesia. An in-situ posterior instrumentation with screws and decompression was performed. Transpedicular screws were placed and the dislocation was corrected using rod including laminectomy of the T8 to T9 vertebrae. However, during rod contouring and fixation, some degree of spontaneous kyphosis correction was achieved passively due to the realignment effect of the rod construct. No kyphosis reduction technique or rib fracture intervention was performed. Three-level fixation was selected to manage the significant mechanical instability, along with associated posterior element and rib fractures. Although the fracture involved a single, non-junctional level, the severity of structural disruption warranted an extended fixation construct to ensure optimal spinal stability, pain relief, and prevention of long-term complications. The surgery was well-tolerated, and there was no postoperative neurological deterioration. The patient was mobilized with the help of a dorsolumbar rigid orthosis on the fourth postoperative day. Postoperative radiographs (Figure 4) showed normal alignment of the thoracic spine, with local kyphosis reduced to 12 degrees postoperatively.

The patient remained hospitalized for 20 days postoperatively and was discharged with appropriate follow-up instructions. During subsequent follow-up visits after 1 month, her neurological status remained intact. Pre-operatively, her pain was rated at 7 out of 10 on the Numeric Rating Scale (NRS). Following surgery, her pain decreased to 4 out of 10 during early follow-up at 1 month and continued to improve, with minimal or no pain reported at both the 2-month and 6-month follow-up evaluations.

## DISCUSSION

The thoracic spine's stability, attributed to its kyphotic curve, rib cage support, and surrounding musculature, typically necessitates a significant force, such as rotational or shear, to result in an injury.^2^ Thoracic spine fractures are distinct from other types of spinal fractures. They are less common than thoracolumbar or lumbar fractures and typically occur around the apex of the thoracic kyphosis, most often between T6 and T9.^6^ Although the thoracic spine is stable, fractures in this region, particularly translational ones, can result in spinal cord injuries due to the narrow spinal canal, often leading to severe neurological deficits.^1^ The Thoracolumbar (TL) Spine Injury Classification System is based on three primary categories adapted from the original Magerl AO concept: Type A (compression), Type B (tension band), and Type C (displacement) injuries. Type A injuries encompass four subtypes: wedge-impaction, split-pincer, incomplete burst, and complete burst. Type B injuries are categorized into purely osseous and osseo-ligamentous disruptions. Type C injuries are further divided into three subtypes: hyperextension, translation, and separation, without additional subgroup divisions.^7^ Thoracic fracture- dislocations with neural sparing can occur when the middle column separates from the posterior column.^8-10^ They may go unnoticed without neurological deficits, particularly if overshadowed by other injuries. High- velocity trauma, such as motor vehicle collisions, is a common cause. Imaging, preferably computed tomography, should include adjacent vertebrae to assess osseous extension, especially for surgical planning.^9^

Jiang et al. (2014) reported fourteen cases, highlighting that fracture-dislocation of the thoracic vertebrae without neurological symptoms is uncommon. However, they noted that, in rare instances, severe thoracic spinal fracture-dislocations can occur without resulting in neurological deficits.^10^ The extent of spinal cord injury is directly influenced by the degree of cord compression caused by the injury. Since the primary treatment for cord injury involves reducing compression through decompression, any event at the injury site that leads to natural decompression during the trauma may result in minimal neurological deficits.^3,11^

In our case, the most probable explanation for the absence of neurological deficits in the translational fracture at T8-T9 could be that the pedicle fractures cause a separation between the posterior and middle columns, which prevents cord injury by maintaining the integrity of the spinal canal. Although the patient was neurologically intact pre-operatively, she presented with progressive back pain persisting for over a month. Imaging revealed possible spinal canal narrowing and compression from retropulsed fracture fragments, despite the presence of a posterior element fracture. While posterior neural arch fractures can often result in auto-decompression, this mechanism is not always reliable, particularly in cases of persistent pain or when there is concern for irritation of neural structures. Given these findings, a posterior approach with laminectomy at the T8-T9 level was performed to achieve adequate decompression, address potential dural irritation, and alleviate the patient's symptoms. Despite being on dorsolumbar immobilization, the patient had persistent pain, which significantly improved after surgical stabilization. This highlights the added benefit of surgery in providing mechanical stability and effective pain relief when bracing alone is insufficient.

In this case, the translational fracture was likely missed due to insufficient adherence to fundamental trauma assessment protocols. As per ATLS guidelines, thorough clinical examination, including log rolling and palpation along the entire spine, remains essential, regardless of imaging availability.^12^ The standard trauma series omits thoracolumbar spine imaging, and without a high index of suspicion guided by clinical findings, such injuries can be overlooked. This underscores the importance of systematic clinical evaluation in trauma care to guide appropriate imaging and avoid missed diagnoses.

## CONCLUSIONS

Translational thoracic spine fractures with intact neurology as in this case, is rare and can lead to missed diagnosis. A high index of suspicion is essential, supported by thorough clinical examination per ATLS guidelines, including spine palpation and log rolling. Standard trauma imaging often omits the thoracolumbar region, underscoring the need for clinical vigilance. MRI is crucial for evaluating neural compromise. Early posterior stabilization and decompression can yield good outcomes, even in neurologically intact patients. Adherence to trauma protocols and appropriate imaging must be standard to prevent oversight of such injuries.
